# Effect of DLBS1033 on Functional Outcomes for Patients with Acute Ischemic Stroke: A Randomized Controlled Trial

**DOI:** 10.1155/2021/5541616

**Published:** 2021-04-07

**Authors:** Rizaldy Taslim Pinzon, Raymond R. Tjandrawinata, Vincent Ongko Wijaya, Vanessa Veronica

**Affiliations:** ^1^Faculty of Medicine, Duta Wacana Christian University, Yogyakarta, Indonesia; ^2^Bethesda Hospital, Yogyakarta, Indonesia; ^3^Dexa Laboratories of Biomolecular Science, Dexa Medica Group, Cikarang, Indonesia

## Abstract

**Background:**

There are still some unmet needs for stroke management and safety. DLBS1033 is a protein fraction extracted from the earthworm *Lumbricus rubellus* that has shown fibrinolytic and fibrinogenolytic activities, reduces blood viscosity, and inhibits platelet aggregation that it can be considered an add-on therapy and potential medical breakthrough in acute ischemic stroke management.

**Objective:**

This study is aimed at measuring the benefit of DLBS1033 in acute ischemic stroke management.

**Methods:**

This was a randomized, open-label trial at a referral stroke center from November 2019 to December 2020. Subjects who met the inclusion criteria were randomly divided into a control group and an experimental group. The control group received standard therapy consisting of aspirin 100 mg once daily, atorvastatin 20 mg once daily, and vitamin B_12_ 100 mg three times daily. The experimental group received standard therapy and DLBS1033 three times daily. The functional outcomes were measured using the National Institutes of Health Stroke Scale (NIHSS), Barthel Index (BI), and modified Rankin Scale (mRS) at baseline, hospital discharge, and day 30.

**Results:**

Collected data from 180 subjects was analyzed. The NIHSS scores' improvements were significantly greater in the experimental group compared to the control group at both hospital discharge (−5.57 ± 2.16 vs. −3.64 ± 2.65; *p* < 0.001) and day 30 (−6.62 ± 2.64 vs. −5.14 ± 2.41; *p* = 0.001). Compared with the control group, the improvements in the BI scores were significantly better in the experimental group, at both hospital discharge (10.69 ± 5.36 vs. 6.64 ± 5.04; *p* < 0.001) and day 30 (10.9 ± 8.19 vs. 8.56 ± 7.45; *p* = 0.003). The distribution of mRS scores was improved in both groups during 30 days of follow-up and was more favorable in the experimental group. In both groups, a favorable outcome (mRS < 2) was achieved better at day 30 (86.7% vs. 80%; *p* = 0.302) than at baseline (0% vs. 6.7%; *p* = 0.028) and at hospital discharge (58.9% vs. 43.3%; *p* = 0.085). There was no clinically significant adverse event related to the study product.

**Conclusions:**

DLBS1033 in addition to the standard care was more effective in improving functional status compared to standard care alone in acute ischemic stroke patients with a similar safety profile.

## 1. Introduction

Stroke is a pathological manifestation of cerebral dysfunction that occurs for longer than 24 hours or induces mortality without indication of causes apart from vascular disorders [1]. Dependent on pathological background, stroke can be categorized into ischemic or hemorrhagic. Ischemic stroke is caused by a disruption of the blood supply to the brain that induces glucose and oxygen depletion in neurons, glia, and vascular cells [2].

Several treatments, including thrombolytic therapy, antiplatelet drugs, anticoagulants, and neuroprotective agents, are widely used to treat ischemic stroke [3]. There are still some unmet needs for stroke management and the safety of these interventions. Thrombolytic agents are not fibrin-specific, causing excessive bleeding [4, 5]. Antiplatelet drugs and anticoagulants prevent platelet aggregation and formation of fibrin strands; these agents can therefore be used to inhibit thrombogenesis but do not affect existing clots [6].

DLBS1033 is lumbrokinase obtained from the extraction of *Lumbricus rubellus* [7]. Lumbrokinase is a collective name for a group of bioactive proteolytic enzymes that has fibrinolytic and fibrinogenolytic activities, reduces blood viscosity, and reduces platelet aggregation [8, 9]. In addition, lumbrokinase is highly specific to fibrin, meaning lumbrokinase does not induce excessive bleeding [10].

This study is aimed at measuring the benefit of DLBS1033 in ischemic stroke patients as add-on therapy. Thus, if DLBS1033 shows a better outcome than standard therapy, DLBS1033 for ischemic stroke patients can be considered an add-on therapy.

## 2. Methods

### 2.1. Study Design and Subject Selection

This study was conducted using a randomized, controlled, open-label design from November 2019 to December 2020. The subjects consisted of acute ischemic stroke patients at Bethesda Hospital Yogyakarta, Indonesia. The inclusion criteria in this study were as follows: (i) male or female, (ii) adult age (>18 years old), (iii) diagnosed for the first time as acute ischemic stroke, (iv) onset of less than 24 hours, (v) not a referral patient, (vi) GCS score of 15, and (vii) NIHSS score less than 14 (mild to moderate severity). The exclusion criteria in this study were as follows: (i) subjects known to have DLBS1033 hypersensitivity, (ii) participated in other studies for the past one month, and (iii) not competent enough in giving approval and answering questionnaires. Subject withdrawal occurs when (i) some serious adverse events were experienced by the subjects, (ii) subjects suffered from any condition that might interfere with medication and assessment, or (iii) subjects died.

### 2.2. Interventions

Subjects who met the inclusion and exclusion criteria will be randomly divided into a control group and an experimental group using a computer-generated randomization program at a 1 : 1 ratio. The control group received standard therapy consisting of aspirin 100 mg once daily, atorvastatin 20 mg once daily, and vitamin B_12_ 100 mg three times daily. The experimental group received standard therapy and DLBS1033: each tablet contains 490 mg of the *Lumbricus rubellus* bioactive fraction with a dosage of three times daily. Both groups received therapy for 30 days.

### 2.3. Outcome Measurement

The main outcomes of this trial were functional status measured using the National Institutes of Health Stroke Scale (NIHSS), Barthel Index (BI), and modified Rankin Scale (mRS) in Indonesian. Functional status was measured at baseline, hospital discharge, and day 30 after initial treatment. The NIHSS is a scoring instrument used to measure the level of stroke-induced impairment; score 0 represents no stroke, and score 42 represents the most severe stroke. The Barthel Index is a scale that calculates disability or dependency in patients with stroke during daily living activities; score 0 represents a fully dependent patient, while score 20 represents an independent patient. The mRS is a 7-point disability scale where 0 is no symptoms at all and 6 indicates the patient is dead.

### 2.4. Statistical Analysis

This study applied intention-to-treat analysis with a 95% confidence interval and 5% type 1 error. The subjects' clinical characteristics were analyzed using univariate analysis. For between-group analysis, categorical variables were assessed using a chi-squared test or Fisher exact test. Meanwhile, for numerical variables, an independent *t*-test or Mann-Whitney *U* test was used, depending on the normality of data distribution. Statistical significance was set at *p* < 0.05. Missing data was calculated using multiple imputations. The analysis was performed using version 21.0 of SPSS.

### 2.5. Ethical Considerations

This research was conducted according to the Declaration of Helsinki. Ethical approval number 07/KEPK-RSB/I/20 was obtained from Bethesda Hospital Yogyakarta, Indonesia. This research has been registered at ClinicalTrials.gov with the clinical trial registry number of NCT04425590.

## 3. Results

**Figure 1:**
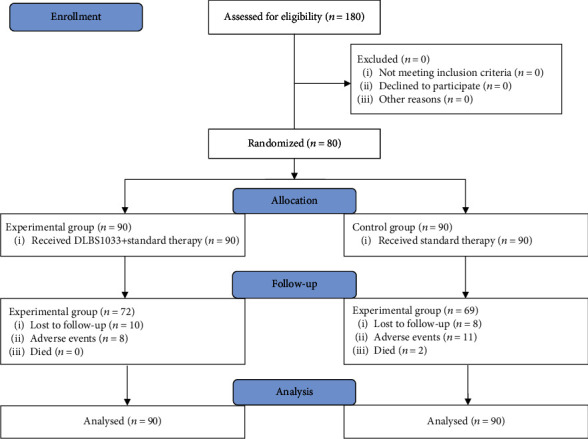
CONSORT flow chart of the study.

A total of 180 eligible subjects were randomly allocated into the experimental group (90 subjects) and the control group (90 subjects). Eighteen subjects in the experimental group (10 subjects were lost to follow-up, and 8 subjects were due to AE) and 21 subjects (8 subjects were lost to follow-up, 11 subjects were due to AE, and 2 subjects died) in the control group terminated the research at the completion of the follow-up, 30 days after initial treatment. Collected data from 180 subjects, including those who terminated the study, were analyzed (CONSORT flow chart; Figure 1).

### 3.1. Subjects' Clinical Characteristics

Subjects' clinical characteristics are shown in Table 1. The number of male and female subjects in this study was the same, namely, 50 (50%) male subjects and 50 (50%) female subjects. The mean age of the subjects was 61.4 ± 10.0 years old in the experimental group and 61.3 ± 11.8 years old in the control group. The most common comorbidity in both groups was hypertension, with a total of 49 (54.4%) subjects in the experimental group and 47 (52.2%) subjects in the control group having hypertension. Eighty-four (93.3%) subjects from the experimental group and 86 (95.6%) subjects from the control group were using antiplatelet drugs, making antiplatelet drugs the most common concomitant medications in this study. The most frequent location and number of lesions on the CT scan findings in this study, respectively, were the subcortex (47.8%) and a single lesion (56.7%). Most of the CT scan findings showed no signs of atrophy (71.1%). The mean scores of the Clock Drawing Test (CDT) in the experimental group and the control group were 2.6 ± 1.5 and 2.2 ± 1.6, respectively. The length of time the subjects were hospitalized ranged from 0 to 24 days with a median of 4 days. There was no significant difference in subjects' baseline characteristics in this study (*p* > 0.05), except for muscle strength (*p* = 0.044).

### 3.2. Laboratory Findings

Table 2 shows the laboratory findings of the subjects. The majority of laboratory findings were within the normal range. Meanwhile, the mean urea numbers of the experimental group and the control group are higher than the normal range. There was no significant difference between both groups in the laboratory findings.

### 3.3. Functional Status Improvement

During the study period, we measured the functional state of subjects in three series with the NIHSS, mRS, and BI in Indonesian. The mean NIHSS scores at baseline, hospital discharge, and day 30 between the experimental group and the control group were 7.3 ± 2.774 vs. 6.02 ± 2.895, 1.73 ± 2.8 vs. 2.38 ± 3.224, and 0.68 ± 2.027 vs. 0.88 ± 2.054, respectively. The comparisons of mRS scores between both groups were 3.48 ± 0.657 vs. 3.1 ± 0.912, 1.54 ± 1.051 vs. 1.92 ± 1.309, and 0.98 ± 0.948 vs. 1.03 ± 1.022, respectively, at baseline, hospital discharge, and day 30. The comparisons of BI scores between both groups at baseline, hospital discharge, and day 30 were 3.96 ± 3.43 vs. 5.48 ± 4.48, 14.64 ± 6.18 vs. 12.12 ± 7.08, and 14.86 ± 8.11 vs. 14.04 ± 8.11, respectively.

**Figure 2:**
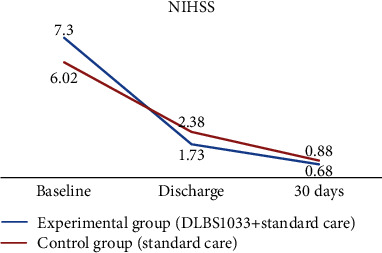
Change from baseline in the mean average National Institutes of Health Stroke Scale (NIHSS) score during the treatment period and follow-up period.

**Figure 3:**
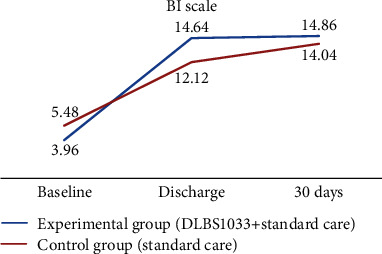
Change from baseline in the mean average Barthel Index (BI) score during the treatment period and follow-up period.

The NIHSS scores' improvements were significantly greater in the experimental group compared to the control group at both hospital discharge (−5.57 ± 2.16 vs. −3.64 ± 2.65; *p* < 0.001) and day 30 (−6.62 ± 2.64 vs. −5.14 ± 2.41; *p* < 0.001). Compared with the control group, the improvements in the BI scores were significantly better in the experimental group, at both hospital discharge (10.69 ± 5.36 vs. 6.64 ± 5.04; *p* < 0.001) and day 30 (10.9 ± 8.19 vs. 8.56 ± 7.45; *p* < 0.001). The NIHSS and BI scores' improvements are shown in Table 3 and Figures 2 and 3.

**Figure 4:**
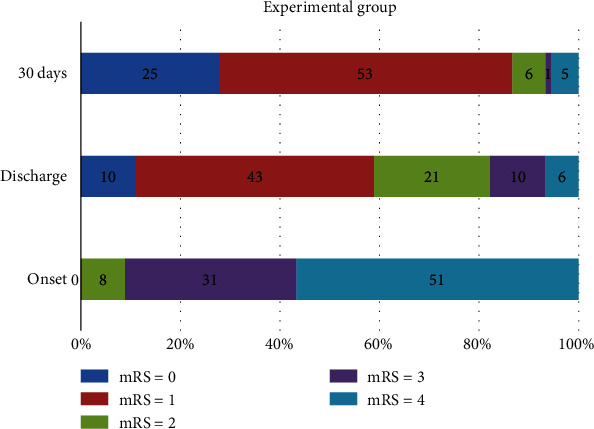
Modified Rankin Scale score distribution at baseline onset, discharge, and day 30 of stroke in the experimental group (DLBS1033 therapy+standard treatment).

**Figure 5:**
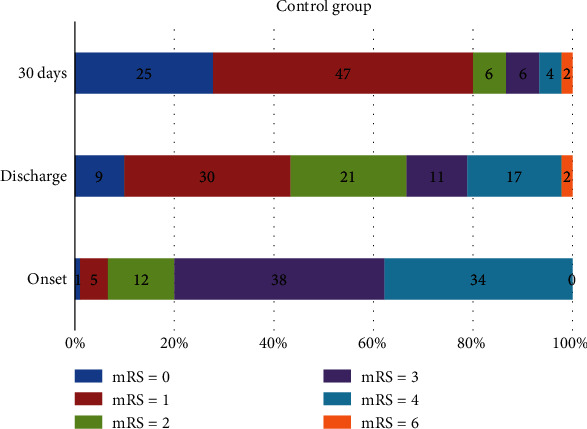
Modified Rankin Scale score distribution at baseline onset, discharge, and day 30 of stroke in the control group (standard treatment only).

Table 4 and Figures 4 and 5 show the proportion of patients at each mRS score in the experimental and control groups at baseline, hospital discharge, and 30 days. There was a significant difference in the distribution of the mRS scores between both groups at baseline (*p* = 0.028). Meanwhile, the distribution of the mRS scores between both groups at hospital discharge (*p* = 0.104) and day 30 (*p* = 0.390) has no significant differences. The mRS scores of <2 were defined as a favorable outcome.

At baseline onset, most of the patients had a less favorable outcome (mRS ≥ 2). In the experimental group, 90 patients (100%) presented with mRS scores more than or equal to 2; in the control group, 84 (93.3%) patients had a less favorable outcome.

Fifty-three patients (58.9%) in the experimental group and 39 patients (43.3%) in the control group had mRS scores of <2 at hospital discharge. The number of subjects who had mRS scores of <2 at day 30 was greater than at hospital discharge. At day 30, patients in the experimental group had a higher rate of favorable outcomes (mRS < 2) compared with the control group (86.7% vs. 80%; *p* = 0.302).

### 3.4. Adverse Events

Tables 5 and 6 compare the adverse events between the experimental group and the control group. There were 14 (8%) subjects experiencing adverse events at hospital discharge, consisting of 6 subjects of the experimental group and 8 subjects of the control group. Five (3.4%) subjects, including 2 subjects of the experimental group and 3 subjects of the control group, experienced adverse events at day 30. Gastrointestinal discomfort, heartburn, vomiting, gastrointestinal tract bleeding, cephalgia, thrombocytopenia, and anemia were the side effects encountered by the subjects (as shown in Table 6); no side effects have been associated with DLBS1033. Approaches taken to manage adverse events were as follows: drug withdrawn, administration of gastroprotective agents (PPIs), gastric lavage, and blood transfusions.

## 4. Discussion

DLBS1033, as protein extract from the earthworm *Lumbricus rubellus*, has been known for its antithrombotic and thrombolytic effects [7]. The present study was carried out to investigate the potential effects of DLBS1033 as treatment of acute ischemic stroke in our trial settings.

Compared with standard treatment only, DLBS1033 administration demonstrated more favorable effects on NIHSS severity, BI functional outcomes, and mRS disability at discharge and at 30 days compared with standard treatment in patients with acute ischemic stroke. DLBS1033 administration versus standard treatment did not exhibit major adverse events with similar safety profiles. A multicenter study reported that the fibrinogen-depleting agent lumbrokinase showed a favorable effect in reducing the volume of atherosclerotic plaques, NIHSS scores, and cerebral vascular events as it was also beneficial for secondary ischemic stroke prevention [11, 12].

This effect was also similar between groups, including age, gender, imaging classification, comorbidities, cognitive status, length of stay, baseline cholesterol, and blood glucose, including other laboratory values. Additionally, DLBS1033 was well tolerated in the current study with no major safety concerns. Gastrointestinal tract-related symptoms were the most commonly reported adverse event in patients treated with either DLBS1033 or standard treatment alone but were similar in frequency for both study arms. Multiple studies also showed that DLBS1033 was proven safe without severe adverse events related to the administration [7, 13].

An elevated fibrinogen level has been known as a major risk factor for ischemic cerebrovascular events. The potential effects of DLBS1033 on acute ischemic stroke have been reported as fibrinolytic, fibrinogenolytic, and antiplatelet aggregation activities. A recent study also showed that the lumbrokinase mechanism of inhibition of the intrinsic coagulation pathway induces fibrinolytic activity by increasing the tPA activity and plasminogen activator. Blood viscosity is related to the fibrinogen level. Thus, the degradation activity of DLBS1033 on the fibrinogen chains can decrease the blood viscosity level. The crucial role of platelet aggregation inhibition by lumbrokinase was exerted by increasing the cAMP level and inhibiting the Ca transport [14, 15]. A meta-analysis of 727 ischemic stroke patients showed a significant reduction in the fibrinogen level and blood viscosity in the lumbrokinase-treated group [16].

However, the effects of DLBS1033 were not immediately observed, unlike intravenous thrombolytic therapy, which can be seen right after use. Therefore, the use of oral DLBS1033 is not recommended to replace the function of intravenous thrombolysis or rtPA in acute ischemic stroke treatment. However, the use of the lumbrokinase agent may be effective as an adjuvant drug alongside the standard treatment for ischemic stroke [11]. A study of oral lumbrokinase efficacy in patients with stable angina pectoris showed that the drug might improve myocardial perfusions and also provide potential benefits for a long period of use in cardiovascular events [17]. The findings of this trial may serve as a ground for further studies for the use of DLBS1033 in reducing morbidity and mortality in ischemic stroke patients.

There are some limitations of the present study regarding the potential effect of the DLBS1033 agent. The effect of DLBS1033 was not studied and compared with different doses. This may raise the question of whether greater effectiveness might be observed with higher DLBS1033 doses. Further studies with a longer treatment period at various doses are needed. Bias due to the study's unblinding nature and lack of a placebo arm may also affect the outcome data. The current study was a hypothesis-generating, exploratory study, and the findings should be validated in future confirmatory studies with a double-blind, placebo-controlled design. Finally, the favorable and safety profile of DLBS1033 compared with standard care in this study is an additional modification for future studies that will allow for a better understanding of the potential efficacy and safety of DLBS1033 with respect to ischemic stroke or other diseases.

## 5. Conclusion

This trial showed that DLBS1033 was more effective in improving functional status compared to standard care only in acute ischemic stroke patients with a comparable safety profile. Our study may support the use of DLBSS1033 as an adjuvant treatment for patients with acute ischemic stroke.

## Figures and Tables

**Table 1:** Subjects' clinical characteristics.

Characteristics	No. (%)			*p* value
	Experimental group (*n* = 90)	Control group (*n* = 90)	Total (*n* = 180)	
Age, mean (SD) (years)	61.4 (10.0)	61.3 (11.8)	62.4 (11.0)	0.24
Gender				
Male	54 (60%)	54 (60%)	108 (60%)	1
Female	36 (40%)	36 (40%)	72 (40%)	
Marriage				
Married	76 (84.4%)	73 (81.1%)	149 (82.8%)	0.645
Divorced	11 (12.2%)	15 (16.7%)	26 (14.4%)	
Not married	3 (3.3%)	2 (2.2%)	5 (2.8%)	
Education				
Elementary school	18 (20%)	14 (15.6%)	32 (17.8%)	0.901
Junior high school	13 (14.4%)	12 (13.3%)	25 (13.9%)	
Senior high school	25 (27.8%)	30 (33.3%)	55 (30.6%)	
Bachelor degree	21 (23.3%)	20 (22.2%)	41 (22.8%)	
Others	13 (14.4%)	14 (15.6%)	27 (15%)	
Occupation				
Civil servant	7 (7.8%)	4 (4.4%)	11 (6.1%)	0.138
Entrepreneur	14 (15.6%)	6 (6.7%)	20 (11.1%)	
Private employee	10 (11.1%)	10 (11.1%)	20 (11.1%)	
Retired	18 (20%)	28 (31.1%)	46 (25.6%)	
Unemployment	14 (15.6%)	21 (23.3%)	35 (19.4%)	
Others	27 (30%)	21 (23.3%)	48 (26.7%)	
Type of health financing				
Public insurance	64 (71.1%)	64 (71.1%)	128 (71.1%)	0.498
Private insurance	1 (1.1%)	0 (0%)	1 (0.6%)	
Fee for service	23 (25.6%)	21 (23.3%)	44 (24.4%)	
Company insurance	2 (2.2%)	5 (5.6%)	7 (3.9%)	
Comorbidities				
Hypertension	49 (54.4%)	47 (52.2%)	96 (53.3%)	0.765
Diabetes mellitus	30 (33.3%)	30 (33.3%)	60 (33.3%)	1
Cardiovascular disease	19 (21.1%)	20 (22.2%)	39 (21.7%)	0.856
Gastrointestinal disease	3 (3.3%)	8 (8.9%)	11 (6.1%)	0.12
Others	4 (4.4%)	7 (7.8%)	11 (6.1%)	0.351
Concomitant medications				
Antihypertensive medication	45 (50%)	45 (50%)	90 (50%)	1
Antidiabetic medication	29 (32.2%)	30 (33.3%)	59 (32.8%)	0.874
Antiplatelet	84 (93.3%)	86 (95.6%)	170 (94.4%)	0.515
PPI/H2 blocker	2 (2.2%)	1 (1.1%)	3 (1.7%)	1
Anticoagulant	0 (0%)	0 (0%)	0 (0%)	1
Muscle strength				
0-no contraction	2 (2.2%)	0 (0%)	2 (1.1%)	0.044
1-visible muscle contraction without limb movement	4 (4.4%)	1 (1.1%)	5 (2.8%)	
2-active movement, but not against gravity	12 (13.3%)	10 (11.1%)	22 (12.2%)	
3-active movement against gravity	26 (28.9%)	15 (16.7%)	41 (22.8%)	
4-active movement against gravity and resistance	43 (47.8%)	55 (61.1%)	98 (54.4%)	
5-normal muscle power	3 (3.3%)	9 (10%)	12 (6.7%)	
Location of lesions on CT scan				
Cortex	32 (35.6%)	29 (32.2%)	61 (33.9%)	0.912
Subcortex	42 (46.7%)	44 (48.9%)	86 (47.8%)	
Cortex and subcortex	16 (17.8%)	17 (18.9%)	33 (18.3%)	
Atrophy on CT scan				
Visible atrophy	24 (26.7%)	28 (31.1%)	52 (28.9%)	0.48
No atrophy	66 (73.3%)	62 (68.9%)	128 (71.1%)	
Number of lesions on CT scan				
Single	53 (58.9%)	49 (54.4%)	102 (56.7%)	0.504
Multiple	37 (41.1%)	41 (45.6%)	78 (43.3%)	
CDT, mean (SD)	2.6 (1.5)	2.2 (1.6)	2.4 (1.5)	0.229
Length of stay, median (range) (days)	4 (0-16)	3 (2-13)	4 (0-24)	0.15

Data are presented as mean ± standard deviation (SD) and *n*(%). Abbreviations: CDT: Clock Drawing Test; CT: computed tomography; GCS: Glasgow Coma Scale.

**Table 2:** Laboratory findings of subjects.

Laboratory finding	Median (IQR)			*p* value
	Experimental group (*n* = 90)	Control group (*n* = 90)	Total (*n* = 90)	
Hemoglobin (g/L)	145.5 (99.0-184.0)	139.0 (99.0-184.0)	138.0 (80.0-184.0)	0.547
Leucocyte count (×10^9^/L)	8.2 (5.4-12.8)	8.0 (5.3-19.2)	8.8 (3.7-20.4)	0.895
Platelet count (×10^9^/L)	258.5 (124.0-502.0)	235.0 (144.0-1291.0)	263.0 (97.0-1291.0)	0.181
Total cholesterol (mmol/L)	4.8 (2.8-8.05)	5.1 (3.3-7.8)	5.1 (2.7-8.1)	0.409
Urea (mmol/L)	10.9 (5.5-40.5)	9.9 (4.4-32.0)	9.9 (4.3-40.5)	0.676
Creatinine (*μ*mol/L)	90.6 (56.6-388.1)	88.4 (41.6-160.9)	85.8 (41.6-388.1)	0.826
Sodium (mmol/L)	139.4 (125.9-150.7)	139.4 (129.9-145.0)	139.9 (125.9-150.7)	0.656
Potassium (mmol/L)	3.9 (2.9-5.9)	3.9 (2.7-5.2)	3.8 (2.7-5.9)	0.858
Blood glucose (mmol/L)	7.8 (2.6-41.9)	7.6 (4.4-18.8)	7.8 (2.6-41.9)	0.665

**Table 3:** Comparison of NIHSS and BI scores between the experimental and control groups.

	Experimental group (*n* = 90)	Control group (*n* = 90)	*p* value
NIHSS			
Baseline	7.3 (2.774)	6.02 (2.895)	0.001
Hospital discharge	1.73 (2.8)	2.38 (3.224)	0.134
Day 30	0.68 (2.027)	0.88 (2.054)	0.278
Change from baseline to hospital discharge	-5.57 (2.16)	-3.64 (2.65)	<0.001
Change from baseline to day 30	-6.62 (2.64)	-5.14 (2.41)	0.001
BI			
Baseline	3.96 (3.43)	5.48 (4.48)	0.047
Hospital discharge	14.64 (6.18)	12.12 (7.08)	0.008
Day 30	14.86 (8.11)	14.04 (8.11)	0.163
Change from baseline to hospital discharge	10.69 (5.36)	6.64 (5.04)	<0.001
Change from baseline to day 30	10.9 (8.19)	8.56 (7.45)	0.003

Abbreviations: SD: standard deviation; BI: Barthel Index; NIHSS: National Institutes of Health Stroke Scale. Data are presented as mean (standard deviation).

**Table 4:** The mRS score distribution.

	No. (%)			*p* value
	Experimental group (*n* = 90)	Control group (*n* = 90)	Total (*n* = 180)	
Baseline				
0-no symptoms at all	0 (0%)	1 (1.1%)	1 (0.6%)	0.028
1-no significant disability despite symptoms	0 (0%)	5 (5.6%)	5 (2.8%)	
2-slight disability	8 (8.9%)	12 (13.3%)	20 (11.1%)	
3-moderate disability	31 (34.4%)	38 (42.2%)	69 (38.3%)	
4-moderately severe disability	51 (56.7%)	34 (37.8%)	85 (47.2%)	
5-severe disability	0 (0%)	0 (0%)	0 (0%)	
6-dead	0 (0%)	0 (0%)	0 (0%)	
Hospital discharge				
0-no symptoms at all	10 (11.1%)	9 (10%)	19 (10.6%)	0.085
1-no significant disability despite symptoms	43 (47.8%)	30 (33.3%)	73 (40.6%)	
2-slight disability	21 (23.3%)	21 (23.3%)	42 (23.3%)	
3-moderate disability	10 (11.1%)	11 (12.2%)	21 (11.7%)	
4-moderately severe disability	6 (6.7%)	17 (18.9%)	23 (12.8%)	
5-severe disability	0 (0%)	0 (0%)	0 (0%)	
6-dead	0 (0%)	2 (2.2%)	2 (1%)	
Day 30				
0-no symptoms at all	25 (27.8%)	25 (27.8%)	50 (27.8%)	0.302
1-no significant disability despite symptoms	53 (58.9%)	47 (52.2%)	100 (55.6%)	
2-slight disability	6 (6.7%)	6 (6.7%)	12 (6.7%)	
3-moderate disability	1 (1.1%)	6 (6.7%)	7 (3.9%)	
4-moderately severe disability	5 (5.6%)	4 (4.4%)	9 (5.0%)	
5-severe disability	0 (0%)	0 (0%)	0 (0%)	
6-dead	0 (0%)	2 (2.2%)	2 (1%)	

Abbreviations: mRS: modified Rankin Scale.

**Table 5:** Adverse events.

	No. (%)		
	Experimental group (*n* = 90)	Control group (*n* = 90)	Total (*n* = 180)
Hospital discharge			
Any adverse event	6 (6.7%)	8 (8.9%)	14 (7.8%)
No adverse event	83 (92.2%)	79 (87.8%)	162 (90%)
Day 30			
Any adverse event	2 (2.2%)	3 (3.3%)	5 (5.6%)
No adverse event	74 (82.2%)	69 (76.7%)	143 (79.4%)

**Table 6:** Type of adverse events.

	No. (%)		
Type of adverse event	Experimental group (*n* = 90)	Control group (*n* = 90)	Total (*n* = 180)
GI discomfort	2 (2.2%)	3 (3.3%)	5 (2.8%)
Heartburn	1 (1.1%)	5 (5.6%)	6 (3.3%)
Vomiting	1 (1.1%)	0 (0%)	1 (0.6%)
GI tract bleeding	4 (4.4%)	3 (3.3%)	7 (3.9%)
Cephalalgia	1 (1.1%)	0 (0%)	1 (0.6%)
Thrombocytopenia	1 (1.1%)	1 (1.1%)	2 (1.1%)
Anemia	0 (0%)	1 (1.1%)	1 (0.6%)

Abbreviations: GI: gastrointestinal.

## Data Availability

Data are available on request from the first author.
